# Profiles of digital disability among Chinese older adults and its association with cognitive function: a latent profile analysis

**DOI:** 10.3389/fnagi.2026.1819606

**Published:** 2026-04-30

**Authors:** Feijia Zhu, Weichao Chen

**Affiliations:** School of Journalism and Communication, Hunan Normal University, Changsha, China

**Keywords:** Chinese, cognitive function, digital disability, older adults, self-perceptions of aging

## Abstract

**Background:**

The relationship between digital disability patterns and cognitive function among older adults remains complex. This research investigates how different patterns of digital disability were associated with cognitive function, with particular attention to the moderating role of self-perceptions of aging.

**Objective:**

This study aims to identify distinct patterns of digital disability among older adults, examine their associations with cognitive function, and investigate whether self-perceptions of aging moderate these relationships.

**Methods:**

Data were collected from the 2020 China Longitudinal Aging Social Survey (CLASS), including 2,848 participants aged 60 and above. Latent Profile Analysis (LPA) was employed to systematically identify distinct digital disability patterns.

**Results:**

The research identified three distinct digital disability patterns: utilitarian digital disability pattern (Class 1, 24.6%), digital empowerment pattern (Class 2, 35.3%), and high digital disability pattern (Class 3, 40.0%). Compared to the Class 2, Class 1 and Class 3 were significantly associated with lower cognitive function, with self-perceptions of aging found to moderate this relationship. Younger older adults ( < 70) and rural residents experienced stronger negative cognitive effects from Class 3 and Class 1.

**Conclusion:**

Targeted digital literacy programs and age-friendly technology designs are essential for maintaining cognitive health in older populations.

## 1 Introduction

Cognitive ability refers to the capacity of the human brain to process, store, and retrieve information (Hunter, 1986). Cognitive decline is a progressive neurodegenerative condition associated with aging among older adults (Kioussis et al., 2021; Li et al., 2019). Increased cognitive decline is likely to cause Alzheimer’s disease, dementia and death (Prince et al., 2015; Shi et al., 2025). By 2018, the number of Alzheimer’s patients in China had risen from approximately 3.68 million in 1990 to nearly 10 million, ranking China as the country with the highest number of cases globally (Liu and Guo, 2022). This trend places significant strain on informal caregiving costs and healthcare resources. Furthermore, the cognitive health of older adults is a crucial component of healthy aging (Mei et al., 2017), emphasizing the importance of early detection of cognitive impairment.

Over the past few decades, the Internet has gradually become integrated into the everyday lives of older adults, transforming modes of communication and social connection (Zhu et al., 2021). The rise in Internet accessibility has led to a gradual increase in Internet use among older adults (World Health Organization [WHO], 2015). Schehl et al. (2019) identified three primary reasons older adults engage in online activities: informational purposes, social purposes, and instrumental purposes. However, older adults face multiple challenges when accessing the Internet. These challenges stemmed from a complex interplay of biological, psychological, and social factors (Xie et al., 2023). Barriers such as limited awareness, access, technical skills, and prior experience often prevented older adults from adopting new technologies (Ge et al., 2025). Furthermore, many older adults lacked confidence in their ability to use technology, which further hindered their adoption (Siren and Knudsen, 2017; Kim et al., 2023). These difficulties contributed to the digital divide, which includes disparities in digital skills, usage, and outcomes (Scheerder et al., 2017). The Internet outcomes digital divide refers to the disparities in how individuals apply digital skills to achieve socio-economic advantages after gaining technological access. Building on this framework, this paper introduced the concept of “digital disability” (Wang, 2024a,Wang, 2024b), which referred to the challenges older adults face in engaging with emerging digital technologies. This limited their access to economic, social, cultural, and health benefits, thus intensifying their marginalization.

Research increasingly showed that digital disability was negatively associated with cognitive functions among older adults. According to the Amplification Theory of Technology, Internet use enabled older adults to both maintain existing relationships and broaden their social networks, which positively influenced their cognitive performance (Sohn et al., 2023; Chen et al., 2024). Li et al. (2024) found that overcoming the digital divide was significantly associated with improved cognitive function, a slower rate of cognitive decline, and a higher likelihood of reverting from MCI to normal cognition. Moreover, longitudinal research indicates that overcoming the digital divide is associated with a decline in the incidence of MCI among older adults after 10 years (Quialheiro et al., 2022).

Given these challenges, developing digital literacy emerges as an important factor in bridging the digital divide and promoting mental health among older adults. The digital literacy of the elderly has been defined as an overarching framework encompassing several interconnected literacies, including skills, knowledge, ethics, and creative expression in the digital environment (de Hoog and Verboon, 2020). Research suggested that digital literacy could help alleviate depression by fostering social connections and increasing access to online mental health resources (Ellison et al., 2007; Hong et al., 2023).

To the best of our knowledge, there are several studies investigating the association between Internet technology usage, online activities, and mental health outcomes. However, previous studies have primarily focused on individual aspects of Internet technology usage and online activities, failing to consider important information within individuals (Chen et al., 2022; Liu et al., 2021), which could be addressed by latent profile analysis (LPA). As a person-centered method, LPA seeks to identify subgroups of individuals who share common characteristics (Hensel, 2020). The classification for clustering individuals in unobserved groups is evaluated using model-based methods and statistical diagnostic tools, ensuring the accuracy and effectiveness of the classification (Muthén and Muthén, 2000). While LPA does not overcome limitations of cross-sectional data, such as the inability to evaluate causal relationships, it enables the identification of subgroups with shared characteristics within a single sample of participants (Ji, et al., 2025).

## 2 Materials and methods

### 2.1 Sample

The China Longitudinal Aging Social Survey (CLASS) is a nationally representative survey conducted by the National Survey Research Center at Renmin University of China. Launched in 2010 and conducted biennially, CLASS examines various aspects affecting older adults, including health, social relationships, and digital technology use.

CLASS utilized a three-stage, probability-proportionate-to-size sampling technique. In the first stage, 134 counties/districts were selected from a comprehensive list of county-level units. In the second stage, 462 villages and communities were randomly selected, maintaining an urban-to-rural ratio of 6:4. Data collection involved face-to-face interviews using standardized questionnaires.

For this study, we analyzed the CLASS 2020 data, which included 11,398 participants. After excluding incomplete or invalid responses, the final sample comprised 2,848 individuals aged 60 and older, offering a representative cross-section of China’s aging population across both rural and urban settings.

### 2.2 Measurement

#### 2.2.1 Dependent variable

This study assessed cognitive function based on the Mini-mental State Examination (MMSE) as provided by CLASS. The instrument encompasses 16 items across four key domains: 5 items assessed orientation, 3 items focused on memory, 5 items measured attention and calculation, and 3 items focused on recall. Responses are scored on a binary scale: 1 point for each correct answer and 0 points for each incorrect answer, resulting in a possible score range of 0–16 points. A higher total score is indicative of superior cognitive functioning.

#### 2.2.2 Independent variable

Consistent with prior research (Wang, 2024a), the study measured the digital disability among the old adults by assessing how prevalent Internet technologies impact eight aspects of their lives: social interaction, shopping and consumption, access to news, leisure and entertainment, travel and tourism, health services, investment and financial management, and learning and training. Each item was rated on a three-point response scale, with 3 indicating “inconvenient,” 2 indicating “no impact,” and 1 indicating “convenient.”

#### 2.2.3 Moderating variable

This study employed self-perceptions of aging as the mediating variable, measured using the Attitudes to Aging Questionnaire (AAQ) developed by Laidlaw et al. (2007). In the CLASS 2020, self-perceptions of aging were assessed through a series of seven statements. Respondents were asked to indicate their level of agreement with each statement using a 5-point scale ranging from “totally disagree” to “totally agree.” The specific questions were: (a) I feel that I am already old; (b) In my opinion, aging is a process of continuous loss (e.g., loss of health, loss of friends and relatives, loss of ability, etc.); (c) I find it more difficult to make new friends as I age; (d) I feel that I am excluded because of my age; (e) The older you get, the better you are at dealing with life’s problems; (f) Wisdom grows with age; (g) There are many pleasurable things about growing old. Each item was rated on a three-point response scale, with the categories: “not at all” = 1, “sometimes” = 2, and “often” = 3. To calculate self-perceptions of aging, scores for statements (a), (b), (c), and (d) were reversed. The total score ranged from 8 to 35, with higher scores indicating a more positive attitude toward aging. The Cronbach’s alpha for this scale was 0.702.

#### 2.2.4 Covariates

Based on previous studies (Lei and Feng, 2021; Sun et al., 2020), the following variables were included as covariates in this study: age, gender, marital status, hukou status, educational level, family annual income, employment status, living arrangement, housing ownership, insurance status, activities of daily living (ADLs), instrumental activities of daily living (IADLs), number of comorbid chronic diseases, social participation, and living region.

### 2.3 Statistical analysis

First, baseline characteristics were summarized as means and standard deviations for normally distributed continuous variables, and as frequencies with percentages for categorical variables. Data were stratified by digital disability patterns and compared using the chi-square test for categorical variables and ANOVA for continuous variables, as appropriate.

Second, we used latent profile analysis (LPA) to identify distinct patterns of digital disability among older adults. As a person-centered method, LPA employs maximum likelihood estimation to assign individuals to groups based on posterior probabilities (Nylund-Gibson et al., 2019). This approach provides more precise classifications than traditional clustering techniques. Such accuracy is crucial when studying older adults, whose digital disability patterns vary significantly depending on factors like education level, urban or rural residence, and prior exposure to technology.

To determine the optimal number of classes, we estimated models with 1–4 latent profiles and evaluated several key fit indices: log-likelihood (LogLik), Akaike’s information criterion (AIC), Bayesian information criterion (BIC), integrated completed likelihood (ICL), and entropy. The selection of the optimal model was guided by established criteria: (1) higher (less negative) values of LogLik and lower values of AIC, BIC, and ICL indicating better model fit, (2) higher entropy values (approaching 1.0) suggesting better classification quality, and (3) significant bootstrap likelihood ratio test (BLRT) results (*p* < 0.01) confirming meaningful improvement in model fit compared to solutions with fewer classes.

Third, Ordinary Least Squares (OLS) regression analysis was employed to examine the relationship between digital disability patterns and cognitive function. Statistical analysis was conducted using the R software version 4.1.0.

### 2.4 Ethical considerations

The datasets generated and analyzed during the current study are publicly available in the China Longitudinal Ageing Social Survey (CLASS) repository, and the authors received permission to use the data. CLASS was approved by the institutional review board at the Renmin University of China, but the ethics approval number has not been publicly released. All procedures performed in the study involving human participants were in accordance with the ethical standards of the institutional or national research committee and with the 1964 Helsinki declaration and its later amendments or comparable ethical standards. All participants provided written informed consent. Details of informed consent were stored by the Institute of Gerontology and National Survey Research Center at Renmin University of China.

## 3 Results

### 3.1 Results of latent profile analysis

Table 1 presents the LPA fit indices from 1-class to 4-class models. We selected the three-class model for the following reasons: Although the four-class model yielded slightly lower AIC (38563.01) and BIC (38819.05) values, the three-class model achieved the highest entropy value (0.95) across all candidate models, indicating superior classification quality and cleaner separation between latent classes. Additionally, the BLRT *p*-values ( < 0.01) confirm the statistical significance of model improvement from the 1-class to the 4-class solution. Overall, the three-class solution demonstrated the best balance between parsimony and classification precision, and was therefore selected as the optimal representation of digital disability patterns among older adults.

**TABLE 1:** Indicators for each digital disability profile in older adults.

Classes	LogLik	AIC	BIC	ICL	Entropy	BLRT_*p*
1	−23930.01	47892.51	47987.78	−47988.01	1.00	–
2	−20685.10	41419.81	41568.67	−41733.02	0.91	<0.01
3	−19654.30	39376.23	39578.67	−39748.22	0.95	<0.01
4	−19239.05	38563.01	38819.05	−39047.12	0.90	<0.01

LogLik, Log-likelihood; AIC, Akaike information criterion; BIC, Bayesian information criteria; ICL, Integrated completed likelihood; Entropy: A measure of classification quality; BLRT, bootstrap likelihood ratio test, *p* < 0.05 suggesting significant better performance.

The model’s statistical robustness is supported by the fit indices presented in Table 1, where the LogLik, AIC, BIC, and ICL values show consistent improvement from the 1-class to 4-class solutions, with high entropy values indicating excellent classification quality, while the BLRT *p*-values ( < 0.01) confirm the statistical significance of the model improvement, collectively supporting the selection of the 3-class solution as the optimal representation of digital disability patterns among the older adults.

**FIGURE 1:**
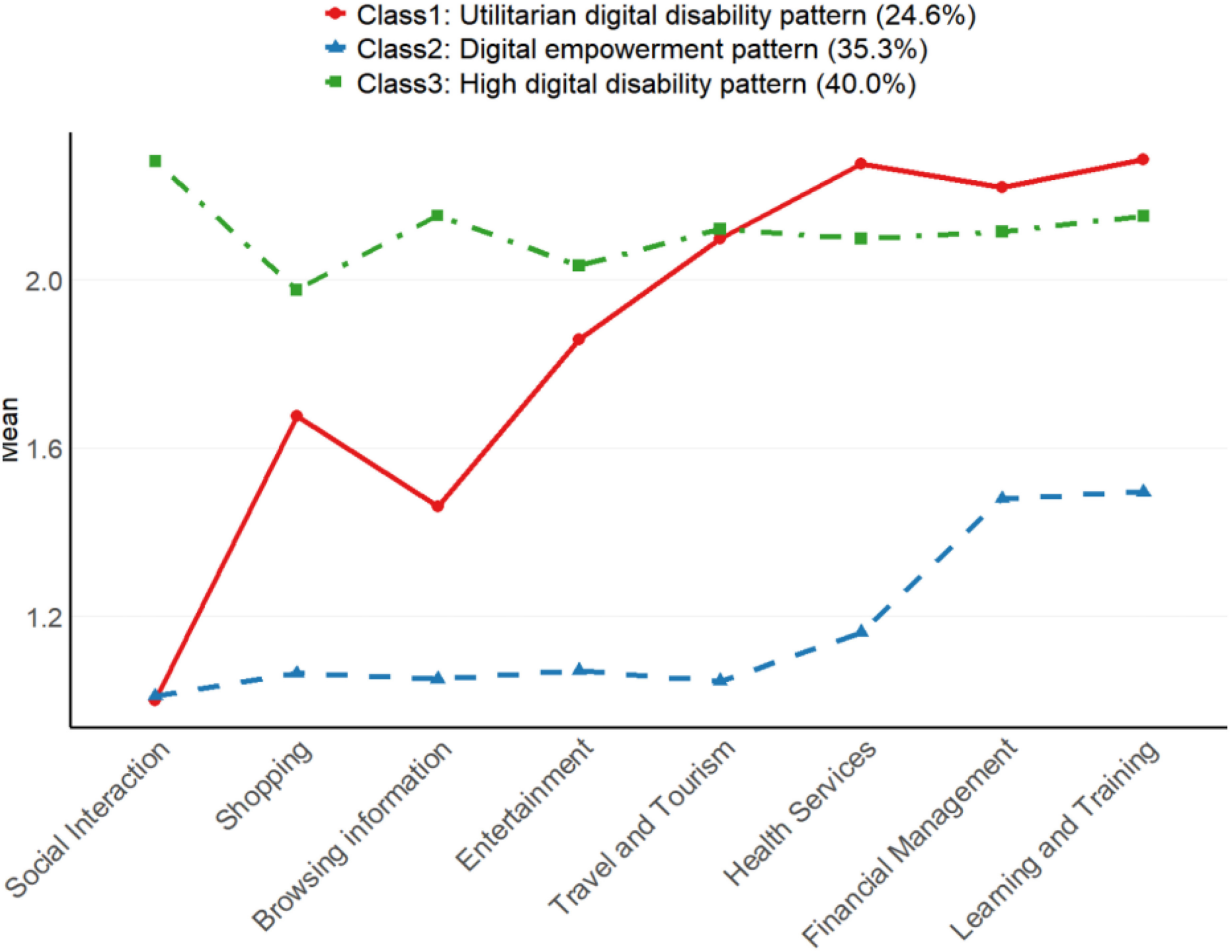
Latent profile model of digital disability.

Based on the Latent Profile Analysis (LPA) conducted to examine digital disability patterns among elderly individuals, three distinct classes emerged from the analysis of eight digital engagement indicators, as illustrated in Figure 1: Class 1 (24.6%) showed a utilitarian digital disability pattern characterized by higher disability in health services, financial management, and learning/training domains, while benefiting more from social interaction and information browsing; Class 2 (35.3%) displayed a digital empowerment pattern with maintaining the lowest disability levels in all categories; Class 3 (40.0%), representing the largest segment, demonstrated an high digital disability pattern with notably higher disability in social interaction, shopping, and entertainment activities.

### 3.2 Sample characteristics

Based on Table 2, the study examined 2,848 elderly participants who were categorized into three distinct digital disability patterns. The overall sample characteristics revealed that participants had a mean depression score of 13.72 (SD = 2.89) and aging attitude score of 20.02 (SD = 3.93), with a balanced gender distribution of 50.3% females and 49.7% males. The average age was 71.24 years (SD = 6.75), with the majority being married (77.0%), not working (79.4%), living with others (93.7%), and having insurance coverage (87.3%).

**TABLE 2:** Baseline characteristics of the total sample and the sample by the different profiles^a^.

Variables	Overall (*n* = 2,848)	Class 1 (*n* = 702)	Class 2 (*n* = 1,006)	Class 3 (*n* = 1,140)	*p* ^b^
Cognitive function, mean (SD)^c^	13.72(2.89)	13.85(2.71)	14.64(2.37)	12.83(3.14)	< 0.001
Self-perceptions of aging, mean (SD)^c^	20.02(3.93)	20.86(4.00)	21.38(3.21)	18.31(3.84)	< 0.001
Gender					0.246
Female	1,433(50.3)	348(49.6)	490(48.7)	595(52.2)	< 0.001
Male	1,415(49.7)	354(50.4)	516(51.3)	545(47.8)	
Age, mean (SD)^c^	71.24(6.75)	71.83(6.87)	69.00(5.81)	72.84(6.90)	
Marital status					< 0.001
Other	656(23.0)	185(26.4)	162(16.1)	309(27.1)	
Married	2,192(77.0)	517(73.6)	844(83.9)	831(72.9)	
Residence					< 0.001
Rural	1,136(39.9)	306(43.6)	272(27.0)	558(48.9)	
Urban	1,712(60.1)	396(56.4)	734(73.0)	582(51.1)	
Educational level					< 0.001
Illiterate	1,136(39.9)	159(22.6)	108(10.7)	315(27.6)	
Literacy class and Primary school	1,712(60.1)	313(44.6)	327(32.5)	516(45.3)	
Middle school and High school	11,36(39.9)	221(31.5)	532(52.9)	292(25.6)	
College and above	1,712(60.1)	9(1.3)	39(3.9)	17(1.5)	
Family annual income, mean (SD)^c^	9.10(0.83)	9.20(0.69)	9.09(0.83)	9.05(0.89)	< 0.001
Current work status					0.001
Not working	2,260(79.4)	511(72.8)	824(81.9)	925(81.1)	
Working	588(20.6)	191(27.2)	182(18.1)	215(18.9)	
Living alone					< 0.001
None	2,669(93.7)	649(92.5)	971(96.5)	1,049(92.0)	
Yes	179(6.3)	53(7.5)	35(3.5)	91(8.0)	
Housing ownership					< 0.001
Not owning a house	16(0.6)	3(0.4)	2(0.2)	11(1.0)	
Owning 1 house	2,409(84.6)	608(86.6)	769(76.4)	1,032(90.5)	
Owning 2 houses and above	423(14.9)	91(13.0)	235(23.4)	97(8.5)	
Insurance					0.013
None	361(12.7)	81(11.5)	110(10.9)	170(14.9)	
Yes	2,487(87.3)	621(88.5)	896(89.1)	970(85.1)	
ADLs, mean (SD)^c^	21.24(2.19)	21.58(1.59)	21.31(2.10)	20.98(2.54)	< 0.001
IADLs, mean (SD)^c^	16.92(2.60)	17.12(2.31)	17.31(2.26)	16.45(2.94)	< 0.001
Number of comorbid chronic disease	1.46(0.72)	1.44(0.70)	1.47(0.72)	1.47(0.73)	0.610
Social participation, mean (SD)^c^.	1.17(2.96)	1.00(2.86)	1.75(3.38)	0.77(2.51)	< 0.001
Living region					0.017
Eastern	1,986(69.7)	470(67.0)	714(71.0)	802(70.4)	
Central	635(22.3)	155(22.1)	226(22.5)	254(22.3)	
Western	227(8.0)	77(11.0)	66(6.6)	84(7.4)	

^a^Data are presented as counts (percentage) unless otherwise indicated.

^b^*P*-value determined using χ^2^ test or analysis of variance *F*-test.

^c^For continuous variables, mean (SD) for each group and significance from analysis of variance F-test are reported. The definition of the classes: Class 1: Utilitarian digital disability pattern, Class 2: Digital empowerment pattern; Class 3: High digital disability pattern.

The digital empowerment pattern (Class 2, 35.3%) demonstrated the highest cognitive ability score and aging attitude score (mean = 21.38). This class was also characterized by the youngest mean age (69.00 years), highest proportion of males (51.3%), highest proportion of married individuals (83.9%), urban residents (73.0%), and highest educational attainment, with 52.9% having middle school and high school education and 3.9% with college education or above. In contrast, the high digital disability pattern (Class 3, 40.0%), representing the largest segment, exhibited the lowest cognitive function scores (mean = 12.83) and having the oldest mean age (72.84 years). This class was also characterized by the highest proportion of females (52.2%), rural residents (48.9%), the lowest educational attainment, and low ADL score (20.98) and IADL score (16.45).

Statistical analysis revealed significant differences (*p* < 0.001) across most variables including cognitive function, aging attitude, age, marital status, residence, educational level, family income, work status, living arrangements, housing ownership, insurance status, ADLs, IADLs, and social participation, while gender distribution showed no significant difference (*p* = 0.246) among the three classes. The number of comorbid chronic diseases also showed no significant difference (*p* = 0.610) across the classes, with the overall sample averaging 1.46 (SD = 0.72) chronic conditions.

### 3.3 Relationship between digital disability patterns and cognitive function

Table 3 examines the relationship between digital disability patterns and cognitive function, with Class 2 (Digital empowerment pattern) serving as the reference group. Multicollinearity was assessed prior to model estimation, and all VIF values were well below the commonly used threshold of 10, with most below 2, suggesting that multicollinearity is not a serious concern in the model.

**TABLE 3:** OLS regression of digital disability patterns on cognitive function (reference: class 2: digital empowerment pattern).

Variable	Model 1	Model 2	Model 3
Class 1	−0.793***(0.137)	−0.337***(0.123)	−0.326***(0.123)
Class 3	−1.814***(0.120)	−1.015***(0.112)	−0.998***(0.112)
Gender (Ref. = Female)		0.010(0.092)	0.016(0.093)
Age		−0.083***(0.009)	−0.085***(0.009)
Marital status (Ref. = Others)		0.229*(0.134)	0.231*(0.134)
Hukou status (Ref. = Rural)		−0.204*(0.115)	−0.149(0.117)
Education (Ref. = Illiterate)			
Literacy class and Primary school		0.512***(0.125)	0.465***(0.127)
Middle school and High school		0.938***(0.146)	0.916***(0.147)
College and above		1.363***(0.329)	1.346***(0.329)
Family annual income		0.094(0.057)	0.113*(0.058)
Employment (Ref. = unemployment)		−0.231*(0.128)	−0.290**(0.131)
Living alone (Ref. = No)		0.067(0.218)	0.044(0.218)
Housing ownership (Ref. = Not owning a house)			
Owning 1 house		−0.199(0.603)	−0.138(0.604)
Owning 2 houses and above		−0.626(0.614)	−0.546(0.616)
Insurance (Ref. = None)		−0.087(0.143)	−0.105(0.142)
ADLs		−0.002(0.034)	−0.004(0.034)
IADLs		0.348***(0.031)	0.344***(0.031)
Number of comorbid chronic disease		−0.119*(0.066)	−0.135**(0.066)
Social participation		0.044***(0.016)	0.043***(0.016)
Living region (Ref. = Western)			
Eastern			−0.221(0.180)
Central			−0.058(0.192)
Adjusted *R*^2^	0.124	0.320	0.321
N	2,848	2,848	2,848

*p < 0.05, **p < 0.01, ***p < 0.001. The definition of the classes: Class 1: Utilitarian digital disability pattern, Class 2: Digital empowerment pattern; Class 3: High digital disability pattern.

Based on Model 3 in Table 3, both Class 1 and Class 3 show significant negative associations with cognitive function (−0.326 and −0.998, respectively, both *p* < 0.001), with Class 3 demonstrating a particularly strong negative relationship. Among sociodemographic factors, higher education levels remain strongly positively associated with cognitive function, with coefficients increasing progressively from primary school (0.465, *p* < 0.001) to middle/high school (0.916, *p* < 0.001) to college and above (1.346, *p* < 0.001). Marital status (0.231, *p* < 0.05), family annual income (0.113, *p* < 0.05), IADL functionality (0.344, *p* < 0.001), and social participation (0.043, *p* < 0.001) show a significant negative association, while age (−0.085, *p* < 0.001), employment status (−0.290, *p* < 0.01), and the number of comorbid chronic diseases (−0.135, *p* < 0.01) were negatively associated with cognitive function. However, gender, Hukou status, living alone, housing ownership, insurance status, ADLs, and region demonstrate no significant associations with cognitive function.

### 3.4 Heterogeneity analyze

Table 4 presents a heterogeneity analysis examining how digital disability patterns affect cognitive function vary across age (<70 vs. ≥ 70) and gender (rural vs. urban) subgroups, using Class 2 (Digital empowerment pattern) as the reference group.

**TABLE 4:** Heterogeneity analysis of the digital disability patterns on cognitive function (reference: class 2: digital empowerment pattern).

Variables	Age		Household	
	<70	≥70	Rural	Urban
Class 1	−0.582***(0.126)	−0.203(0.236)	−1.011***(0.230)	−0.099(0.141)
Class 3	−1.347***(0.118)	−0.821***(0.211)	−1.392***(0.210)	−0.883***(0.130)
*R* ^2^	0.134	0.261	0.271	0.399
N	1,551	1,297	1,136	1,712

OLS estimates are reported; All control variables are controlled. ***p < 0.001. The definition of the classes: Class 1: Utilitarian digital disability pattern, Class 2: Digital empowerment pattern; Class 3: High digital disability pattern.

For age-related differences, Class 1 shows negative associations with cognitive ability only in the < 70 group, while no significant association is observed in the ≥ 70 group. In Class 3, digital disability is significantly associated with lower cognitive performance in both age groups, with a more pronounced effect among those aged < 70 compared to those aged ≥ 70.

In terms of household differences, Class 1 exhibits a significant negative association with cognitive function only among rural older adults, with no significant relationship observed in the urban group. Class 3 shows significant negative associations with cognitive ability in both rural and urban residents, but the association is stronger among rural older adults.

### 3.5 The moderating effect of self-perceptions of aging

Table 5 presents the moderation effect analysis of self-perceptions of aging on digital disability patterns and cognitive function. Both Class 1 and Class 3 show strong negative main effects (−4.236 and −5.051, respectively, *p* < 0.001). The significant positive interaction terms (Interaction 1: 0.214, Interaction 3: 0.184, both *p* < 0.001) reveal self-perceptions of aging ’s crucial moderating role.

**TABLE 5:** The results of the moderation effect analysis (*N* = 2,848).

Variables	Model 1	Variables	Model 1
Class 1	−4.236***(0.686)	Interaction 1	0.214***(0.029)
Class 3	−5.051***(0.609)	Interaction 3	0.184***(0.032)
Self-perceptions of aging	−0.003(0.023)	*R* ^2^	0.351

***p < 0.001. All control variables are controlled.

**FIGURE 2:**
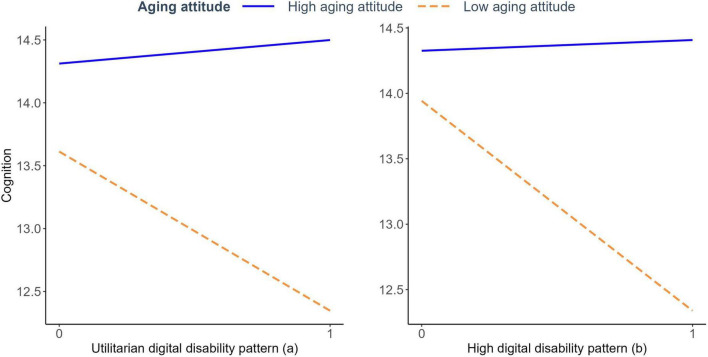
Self-perceptions of aging moderates the relation between digital disability and cognitive function. **(a)** Utilitarian digital disability pattern; **(b)** high digital disability pattern.

Figure 2 visually confirms this relationship, showing divergent patterns between high and low self-perceptions of aging groups. Individuals with positive self-perceptions of aging (blue lines) maintain relatively stable cognitive function regardless of digital disability pattern, while those with negative self-perceptions of aging (orange dashed lines) experience substantial cognitive decline associated with digital disability patterns. This suggests that positive self-perceptions of aging serve as a protective buffer against the negative cognitive impacts of digital disability, with the model explaining 35.1% of the variance.

## 4 Discussion

This study investigated the relationship between digital disability patterns and cognitive function among older Chinese adults using CLASS 2020 data. Further examination revealed distinct characteristics across three digital disability profiles: Class 1 (Utilitarian digital disability pattern, 24.6%), Class 2 (Digital empowerment pattern, 35.3%), and Class 3 (High digital disability pattern, 40.0%). The digital empowerment pattern (Class 2) demonstrated the highest cognitive ability score and aging attitude score, the youngest mean age, highest proportion of males, highest proportion of married individuals, urban residents, and highest educational attainment. In contrast, the high digital disability pattern (Class 3), representing the largest segment, exhibited the lowest cognitive function scores, the oldest mean age, the highest proportion of females, rural residents (48.9%), the lowest educational attainment, and low ADL score and IADL score.

Our findings reveal that patterns of different digital disability patterns have a significant impact on cognitive function. The high digital disability pattern (Class 3) demonstrates a stronger negative association with cognitive function compared to Class 2, followed by the utilitarian digital disability pattern (Class 1). This aligns with the findings of Kamin and Lang (2020), who found that digital inclusion exhibits a positive effect on word, math, memory, number series, and overall cognitive ability test scores among older adults. This may be because digital empowerment enhances cognitive engagement, social connections, and access to information resources that stimulate cognitive ability. These digital disability patterns can be interpreted through the lens of the third-level digital divide (van Deursen and van Dijk, 2023), which concerns not access or skills, but whether digital engagement translates into tangible life outcomes. Our findings suggest that older adults with higher digital disability are systematically unable to convert digital participation into cognitive benefits, reflecting a deeper form of digital exclusion that manifests as inequality in cognitive health outcomes.

Notably, the heterogeneity analysis revealed important demographic variations in the relationship between digital disability patterns and cognitive function. Younger older adults’ (<70) cognition appeared to be more susceptible to the influence of digital disability patterns on cognitive function compared to those aged 70 and above. This aligns with the findings of Köttl et al. (2021) on age-related cognitive decline. Geographically, rural older adults showed consistently stronger associations between digital disability patterns (Class 1 and Class 3) and cognitive function compared to those urban counterparts. This geographic disparity arises from differences in education levels and resource availability between urban and rural areas. Urban elderly typically have higher education levels and greater access to digital resources, which help them better adapt to digital technologies. These resources could enhance digital engagement and provide cognitive stimulation that supports the maintenance and improvement of cognitive function.

Third, self-perceptions of aging played a moderating role in the association between digital disability (Class 3 and Class 1) and cognitive function. Specifically, positive self-perceptions of aging serve as a protective buffer against the negative cognitive effects of digital disability. Zhu et al. (2023) found that negative attitudes toward aging predict cognitive decline.

We propose several policy recommendations: Firstly, improve the age-friendliness of digital products, particularly utilitarian applications related to health services, financial management, and learning. Designers and developers should prioritize creating intuitive interfaces with larger text sizes, simplified navigation, and clear instructions to accommodate the cognitive and physical needs of older users. Secondly, improving the digital literacy of Internet users among older adults, and enhancing their ability to assess online information. The government should integrate the improvement of digital literacy and information literacy of the elderly in the scope of public services, combined with education and community volunteer services. These programs should be tailored to the unique characteristics of older adults, such as limited Internet experience, low awareness of online security risks, and established preferences for certain information sources. Thirdly, provide mental health interventions focused on enhancing self-perceptions of aging, combined with digital literacy training, to cultivate a holistic and empowering approach to digital engagement.

Several limitations of this study should be noted. First, as the data were cross-sectional, it was not possible to dynamically observe changes in the association between digital disability patterns and cognitive function, thereby limiting causal inference and constraining the generalizability of the findings, as such data capture associations at a single point in time. Second, because this study uses secondary data, we are limited to analyzing only the variables that were originally collected, which may not perfectly align with our research objectives.

## 5 Conclusion

This study reveals the intricate relationship between digital disability patterns and cognitive function among older adults in China. We found that the high digital disability pattern has the strongest negative relationship with cognitive function, with notable variations across age groups and household locations. Self-perceptions of aging emerged as a critical moderator, demonstrating that positive attitudes toward aging can buffer the negative cognitive impacts of digital disability.

## Data Availability

Publicly available datasets were analyzed in this study. This data can be found at: Restrictions apply to the availability of these data. Data were obtained from the National Survey Research Center at the Renmin University of China and are available at http://class.ruc.edu.cn/ (accessed on 18 August 2025) with the permission of the National Survey Research Center at Renmin University of China.
